# A dengue outbreak in a rural community in Northern Coastal Ecuador: An analysis using unmanned aerial vehicle mapping

**DOI:** 10.1371/journal.pntd.0009679

**Published:** 2021-09-27

**Authors:** Gwenyth O. Lee, Luis Vasco, Sully Márquez, Julio C. Zuniga-Moya, Amanda Van Engen, Jessica Uruchima, Patricio Ponce, William Cevallos, Gabriel Trueba, James Trostle, Veronica J. Berrocal, Amy C. Morrison, Varsovia Cevallos, Carlos Mena, Josefina Coloma, Joseph N. S. Eisenberg

**Affiliations:** 1 Department of Epidemiology, University of Michigan, Ann Arbor, Michigan, United States of America; 2 Instituto de Geografía, Universidad San Francisco de Quito, Quito, Ecuador; 3 Instituto de Microbiología, Universidad San Francisco de Quito, Quito, Ecuador; 4 Gestión de Investigación, desarrollo e Innovación, Instituto Nacional de Investigación en Salud Pública (INSPI), Quito, Ecuador; 5 Instituto de Biomedicina, Universidad Central del Ecuador, Quito, Ecuador; 6 Department of Anthropology, Trinity College, Hartford, Connecticut, United States of America; 7 Department of Statistics, University of California, Irvine, California, United States of America; 8 Department of Pathology, Microbiology, and Immunology, School of Veterinary Medicince, University of California, San Diego, California, United States; 9 Division of Infectious Diseases and Vaccinology, School of Public Health, University of California, Berkeley, California, United States of America; Centre hospitalier de Cayenne, FRANCE

## Abstract

Dengue is recognized as a major health issue in large urban tropical cities but is also observed in rural areas. In these environments, physical characteristics of the landscape and sociodemographic factors may influence vector populations at small geographic scales, while prior immunity to the four dengue virus serotypes affects incidence. In 2019, a rural northwestern Ecuadorian community, only accessible by river, experienced a dengue outbreak. The village is 2–3 hours by boat away from the nearest population center and comprises both Afro-Ecuadorian and Indigenous Chachi households. We used multiple data streams to examine spatial risk factors associated with this outbreak, combining maps collected with an unmanned aerial vehicle (UAV), an entomological survey, a community census, and active surveillance of febrile cases. We mapped visible water containers seen in UAV images and calculated both the green-red vegetation index (GRVI) and household proximity to public spaces like schools and meeting areas. To identify risk factors for symptomatic dengue infection, we used mixed-effect logistic regression models to account for the clustering of symptomatic cases within households. We identified 55 dengue cases (9.5% of the population) from 37 households. Cases peaked in June and continued through October. Rural spatial organization helped to explain disease risk. Afro-Ecuadorian (versus Indigenous) households experience more symptomatic dengue (OR = 3.0, 95%CI: 1.3, 6.9). This association was explained by differences in vegetation (measured by GRVI) near the household (OR: 11.3 95% 0.38, 38.0) and proximity to the football field (OR: 13.9, 95% 4.0, 48.4). The integration of UAV mapping with other data streams adds to our understanding of these dynamics.

## Author summary

We describe how information collected from unmanned aerial vehicles (drones) can be combined with data from other sources to understand risk factors for dengue during an epidemic that occurred in a small town in Ecuador. The sources we used included data from the Ministry of Health, from entomological surveys, and from community surveillance and questionnaires. We found that people of Afro-Ecuadorian ethnicity were at higher risk for dengue during the outbreak than people of Indigenous ethnicity. This seemed to be because Afro-Ecuadorian households had more nearby vegetation and lived closer to a football field, which may have been a key site for transmission.

## Introduction

Dengue is a major global health concern with over half of the world’s population at risk of infection with dengue virus (DENV) [1]. In 2019 and early 2020, significant dengue epidemics occurred throughout the world, including in Latin America [2], where nearly 3 million cases were reported [3].

Although dengue transmission in rural areas has been extensively described, particularly in Asia, transmission dynamics between and within these sites continues not to be fully understood [4–6]. Recently, however it has been recognized that the burden of rural dengue may be increasing [7] and may even exceed that of urban areas [8]. The transmission dynamics of dengue in rural areas compared to urban ones are likely distinct, not only due to human population size and density but also because the physical environment of these communities may provide different habitats for *Aedes aegypti*, the main vector of DENV [9]. For example, in one study from Vietnam, rainfall was a major driver of dengue in urban areas, while in rural areas *Aedes* production was most prevalent in water storage containers filled by residents [8].

Within rural environments, as in urban ones, the factors driving dengue transmission may also be spatially heterogeneous. *Ae*. *aegypti* has a short range of movement, resulting in extensive clustering in mosquito density between, and within, neighborhoods and households [5,10]. Larval development sites may also migrate within the environment over relatively short periods of time [4], in the form of transient features such as small collections of standing water on floors or plastic tarps, water storage containers, or sites of trash or debris accumulation [11]. Understanding this variability at high spatial resolution is critical to developing effective control mechanisms.

Unmanned aerial vehicle (UAV, i.e. drone) mapping can be used to collect detailed spatial information, with potential applications to the study of vector-borne disease. UAVs have been shown to be useful in identifying the habitats of *Anopheles* mosquitos using spectral analysis to differentiate water bodies that may represent larval habitats [12,13]. UAVs can also be used to characterize land cover, ecological boundaries and transition zones [14], and ancillary features, such as paths or access points, that may be useful for qualitatively understanding the geography of a community, or for logistical planning purposes [13]. To apply UAV mapping to the identification of *Ae*. *aegypti* in human communities, there is interest in identifying larval habitats and density directly [15], but also in characterizing factors such as vegetation that influence local vector populations [16] because they are associated with food, shade and local moisture supply that can reduce evaporation from containers, and decreased sub-canopy wind speed and increased humidity near the ground—all factors that increase vector competence [17–20]. Applications of UAV remote sensing for vegetation mapping have been extensively developed for agriculture and ecosystems management. One of the additional benefits of UAV mapping is that it can be deployed repeatedly, at low cost, to understand how the spatial distribution of the vector may evolve over short time scales, such as weeks or months [4]–thereby complementing remote sensing data.

We combined UAV technology with other data streams to investigate a 2019 dengue outbreak in a rural community in Esmeraldas. Esmeraldas Province in Northwestern Ecuador is among the poorest in the country [21]. About 44% of all residents identify as ‘Afro-Ecuadorian’ and 3% as Indigenous [21], and both groups have historically faced political and social marginalization [22]. These structural inequalities and health disparities may result in greater vulnerability to infectious disease outbreaks [23]. These are further exacerbated when the community is rural and relatively challenging to access [24].

The objectives of this study are: (i) to describe this outbreak, and (ii) to explore the use of UAV mapping as a tool to identify spatial risk factors for symptomatic dengue.

## Methods

### Ethics statement

The study protocol was reviewed and approved by the Universidad San Francisco de Quito (2017-159M) and the University of Michigan (HUM00140967) ethics review boards. It was also reviewed and approved by the Ecuadorian Ministry of Health (Ministerio de Salud Pública, MOH) at the local and the national level (MSPCURI000237-2). Written informed consent was obtained from all adult participants, parental written informed consent was obtained for all children under six years of age, verbal assent and parental written informed consent were obtained for all children 6–8 years old, and written assent and parental written informed consent were obtained for all children 9–17 years old.

### Study site

**Fig 1:**
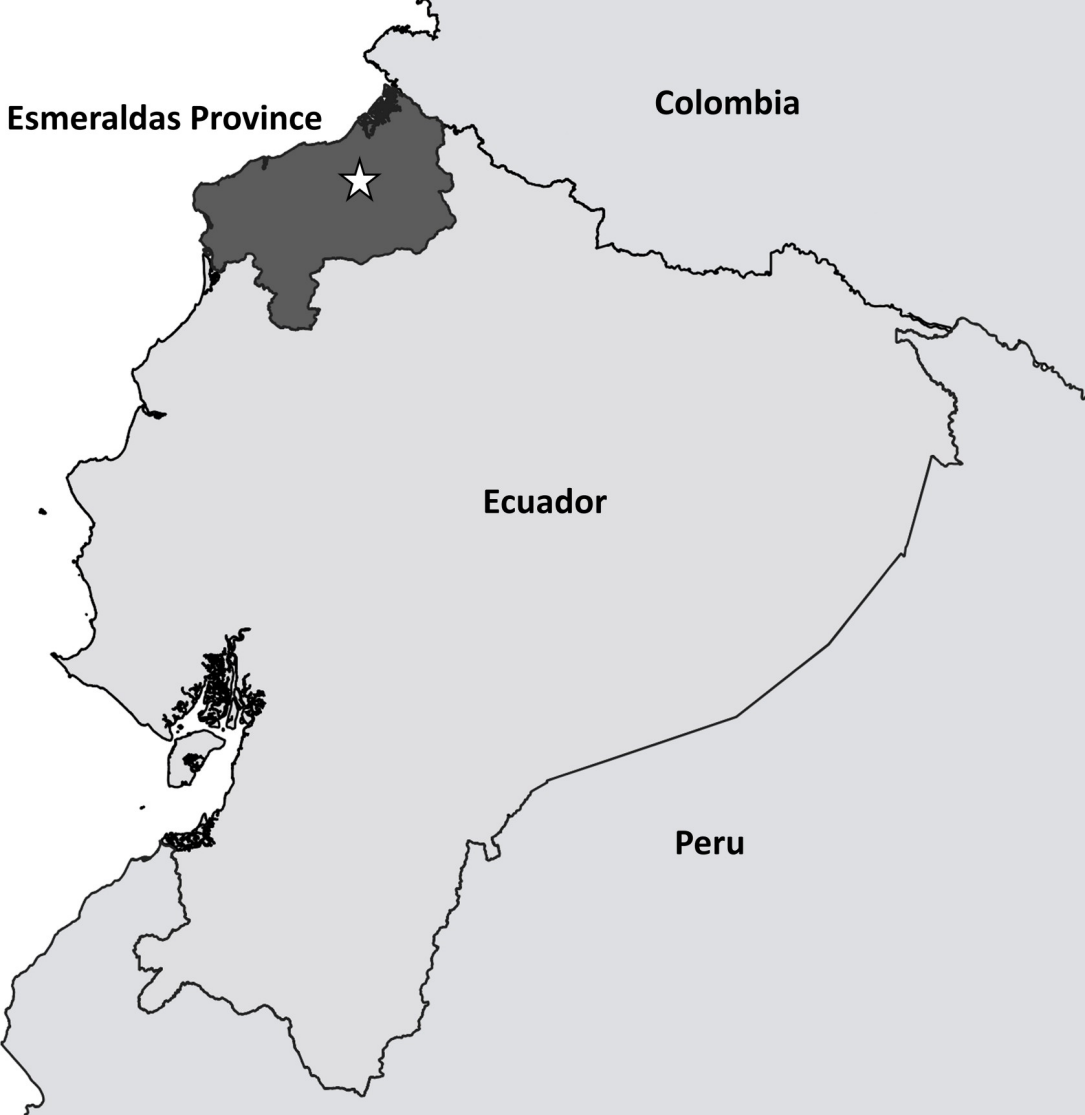
Map of Ecuador: The approximate location of the study community is indicated by the star. This map was constructed using shapefiles downloaded from https://data.humdata.org/, which is available under a CC BY-IGO license (https://data.humdata.org/dataset/ecuador-admin-level-2-boundaries).

**Fig 2:**
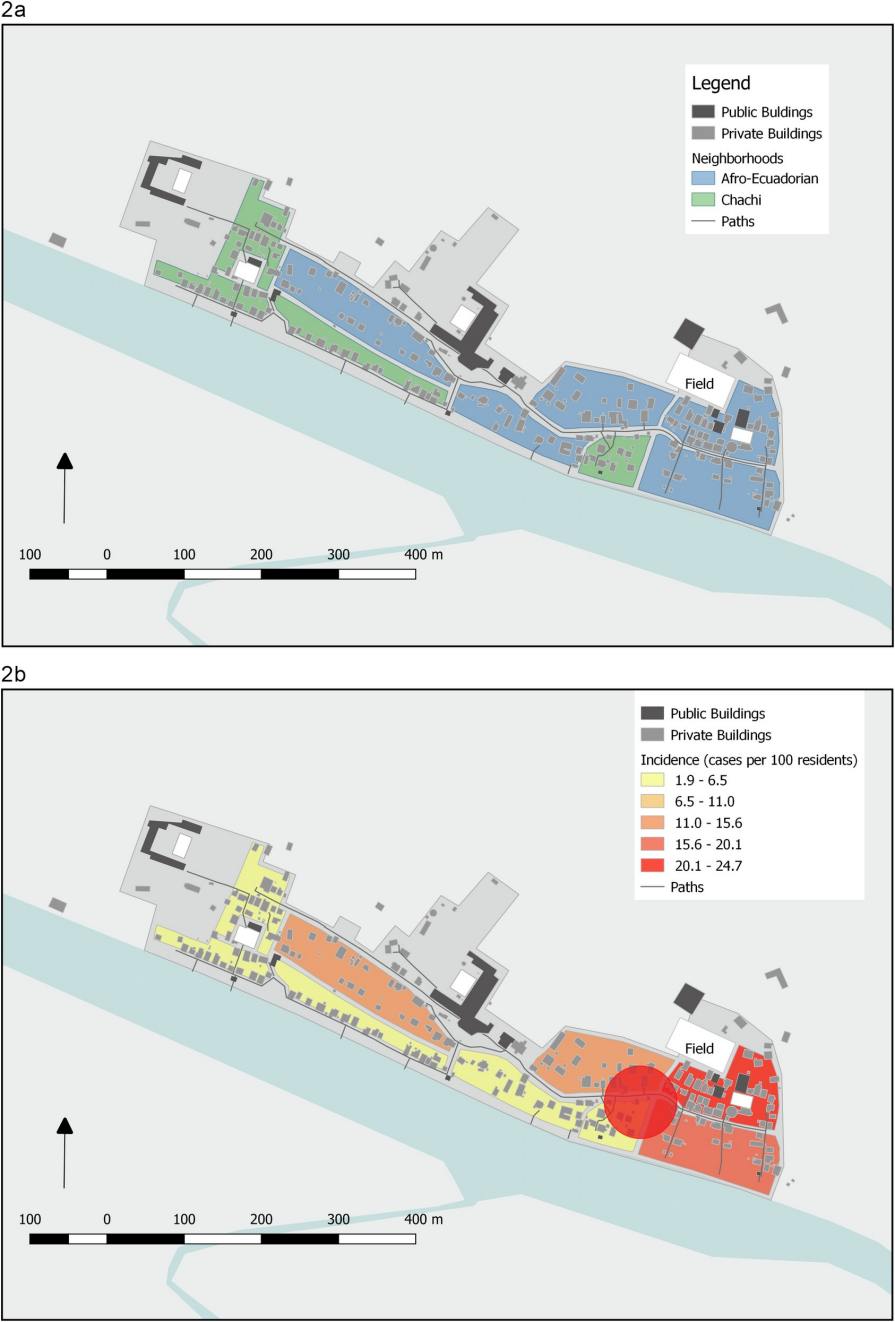
Community Map. The study community. **2a.** Neighborhoods are coded by the reported ethnicity of the occupants. ‘Field’ represents the football field that represented a spatial risk factor in the outbreak. **2b.** The red circle in indicates the part of the community where *Ae. Aegypti* were identified. For privacy reasons, we do not identify specific households where dengue cases occurred, or where *Ae. Aegypti* were identified. These maps were constructed de novo by the study team.

The study village is in the province of Esmeraldas (**Fig 1**) on the bank of a large river. In 2018, it was selected for inclusion into a prospective cohort study of dengue transmission in the region. This study aimed to characterize transmission dynamics across six communities representing a rural to urban gradient. This specific study community, which is about three hours of travel, by boat, from the nearest population center of Borbón, is one of two communities with no road access participating in the overall study. At the time of censusing in 2018, prior to the outbreak, there were 128 inhabited households and 578 residents. Although most communities in this region are either of primarily Afro Ecuadorian or primarily Indigenous Chachi ethnicity, this village is comprised of both ethnic groups (formed through the union of two earlier Afro Ecuadorian and Indigenous communities). Most Afro Ecuadorians live on the eastern side of the village, while most Indigenous Chachis live on the western side (**Fig 2**). While dengue is common in the province Esmeraldas, with an incidence of 53 to 321 per 100,000 residents over the past 5 years [25], most cases occur in the urban centers of the cities of Esmeraldas and San Lorenzo. Prior to the outbreak described here, to the best of our knowledge and per Ministry of Health records, no large dengue outbreaks had been reported in this community or others nearby [25].

As part of this prospective, community-based study to characterize dengue transmission dynamics in the region in both urban and rural areas, several data collection activities were undertaken in the community. These include the following:

### Census

In August 2018, a community census was conducted, during which all households were georeferenced. All individuals in the entire community were invited to participate in the study, and basic demographic information such as age, sex, ethnicity, and livelihoods were collected. In late August and early September of 2019, a follow-up survey was conducted to collect information about household socioeconomic status as well as primary sources of domestic and drinking water.

### Case identification

Dengue cases were identified through a combination of active community surveillance and passive clinic-based surveillance. Active community surveillance data for the study only began in June of 2019, and is therefore available from June-December of 2019, while clinic-based surveillance data are available for all of 2019. Active community surveillance was completed by trained study team of local residents (brigadistas), who included individuals of Afro-Ecuadorian and Chachi ethnicity. Surveys were conducted in Spanish or Chapalaá, the native Chachi language. Brigadistas visited households weekly in the rainy season (June-October), and every other week in the dry season (November-December) to identify reported cases of fever, red eyes, or rash. Brigadistas also identified instances of travel.

If symptoms were reported, a study nurse then visited the household to complete a follow up questionnaire, collect a serum sample and conduct a DengueDUO NS1 and IgM rapid test (Standard Diagnostic Inc., Korea). Convalescent samples were obtained two weeks later during a house visit. Serum samples were stored and transported in liquid nitrogen, to the Universidad San Francisco de Quito, where multiplex dengue, Zika, chikungunya PCR assays [26] and Dengue IgM ELISAS were run. For this analysis, we consider a participant to have had dengue if the case was detected through study active surveillance and was NS1 or IgM positive by rapid test or ELISA, or positive by PCR. Cases were also diagnosed at the local MOH clinic, either with laboratory confirmation (26 cases) or without laboratory confirmation (1 case).

### Vector control activities

Data on vector control activities was shared by the MOH. This included information on the timing, motivation, and completeness of larvicide application in water containers and fumigation campaigns. These activities were conducted both pre-emptively during the rainy season and in response to suspected cases [27].

### Entomological survey

On May 19th, 2019, the study conducted an entomological survey. In a subset of 55 households, containers with water were identified, and *Ae aegypti* immature forms were identified and enumerated. Adult mosquitos were collected with a Prokopack aspirator in domestic and peri-domestic environments. Indoor areas were aspirated for 10 minutes, with an emphasis on the areas where occupants spend most of their time, e.g.: bathrooms, bedrooms, living room and dining room. The peri domestic area immediately outside of the house was aspirated for a further 5 minutes. Aspirated specimens were classified by sex and species on the day of collection and engorged females pooled, frozen and preserved for further detection of DENV using qRT-PCR.

### UAV mapping

Prior to UAV mapping, we reviewed Ecuadorian legal codes surrounding the use of UAVs (Dirección general de aviación civil, resolución N251/2015), sought authorization from local police, and sought the permission of local community leaders. All authorities were informed of the dates during which flights would be made. We also conducted focus groups with residents of a separate study community to understand how the use of drones was likely to be perceived by residents. Results of these focus groups suggested that residents were already familiar with drones, both as children’s toys and in the context of military anti-narcotrafficking activity in the region. Additionally, we learned that if the source of the drone, and reason for the flight, was known, their use was acceptable. UAV mapping was scheduled to coincide with entomological surveys. On May 18^th^ and 19^th^ of 2019, three UAV flights of the community were conducted at 35m altitude. Flight paths were programmed using the Pix4D application (Pix4D SA, Prilly, Switzerland). Orthographic maps were then created using Agisoft Software (Agisoft LLC, St. Petersburg, Russia). The resolution provided by the drone images is comparable to commercially available satellite imagery.

### Statistical methods

To describe this outbreak, we visualized the location of cases over time, as well as the timing of vector control activities undertaken by the local health system in response to the outbreak. We also summarized entomological indices derived near the beginning of the outbreak, in late May.

To identify spatial risk factors for symptomatic dengue, we used UAV images to identify water storage containers by visually inspecting these images. We then used UAV images to calculate the green red vegetation index (GRVI), representing the relative vegetation of the site [28]. Data from households that declined to participate in the study was treated as missing. Based on prior analyses of *Ae*. *aegypti* data from this region [29], we then constructed 40 meter buffers around each household. We used these buffers as the basis for several calculations. Within each buffer, we calculated the mean GRVI and the number of containers visually identifiable in UAV images. We measured whether the household was located within 40m of key public buildings and spaces, such as schools and churches. We calculated Spearman correlation to measure the strength of association between the number of UAV-identified containers per household and the number of containers counted by entomologists during surveillance. Finally, we calculated the local population density as the total number of individuals reportedly living within 40m of the household.

In addition to spatial factors, we also identified potential individual- and household-level risk factors for dengue. At the individual level, we examined age, sex, reported ethnicity, and reported livelihood. Reported livelihoods were collapsed into a smaller number of categories including ‘rural’ work that often requires travel outside of the village (cultivating land, physical labor, or collecting food); employment by the government, such as teaching or working in a health center; small business ownership; domestic work; and other work. For visualization, we summarized reported ethnicity at the household-level, based on the most reported ethnicity of the household. Most households were reported to be either entirely Afro- Ecuadorian or entirely Chachi, however, in the case that both ethnicities were reported in the same household, the most common ethnicity was assigned to that household. Other variables included in the analysis were: 1) the size of the household, 2) the highest completed level of education for any adult in the household, 3) the observed wall, floor, and roof construction material, and 4) the reported primary water source.

To identify individual and household-level risk factors for symptomatic DENV infection, the distributions of individual-level variables such as age, sex, and reported ethnicity were summarized and compared between cases and non-cases using t-tests or chi-squared tests. Based on the observation that Afro-Ecuadorian ethnicity was strongly associated with symptomatic DENV, we further compared household-level characteristics between Afro-Ecuadorian and Chachi homes, including the number of people in the home, construction materials, and reported water sources and water storage practices.

Finally, we constructed logistic regression models. Based on the results of a likelihood ratio test comparing a random-effect model to a model without random effects, we included a household-level random intercept to account for instances when more than one case per household was observed, thus accounting for within household correlation (Chi-bar = 1.97, p = 0.0803). Simple logistic regression models were constructed using each risk factor of interest at a time. All variables significant at the p<0.150 level or greater were then considered for inclusion in a multivariate logistic regression model. Given that several key features of the community, including the football field and the police station, as well as the river and the ‘Piragua’, were located close to each other and each had independent associations with case status (**S2 Table**), we screened covariates by examining the correlation between them. Final model selection was based on comparison of Akaike’s Information Criteria (AIC). We interpreted a reduction in the magnitude of the regression coefficient of ethnicity, after adjusting for spatial risk factors, as evidence that the given spatial covariate partially explained the ethnicity-dengue relationship.

## Results

### Description of the outbreak

**Fig 3:**
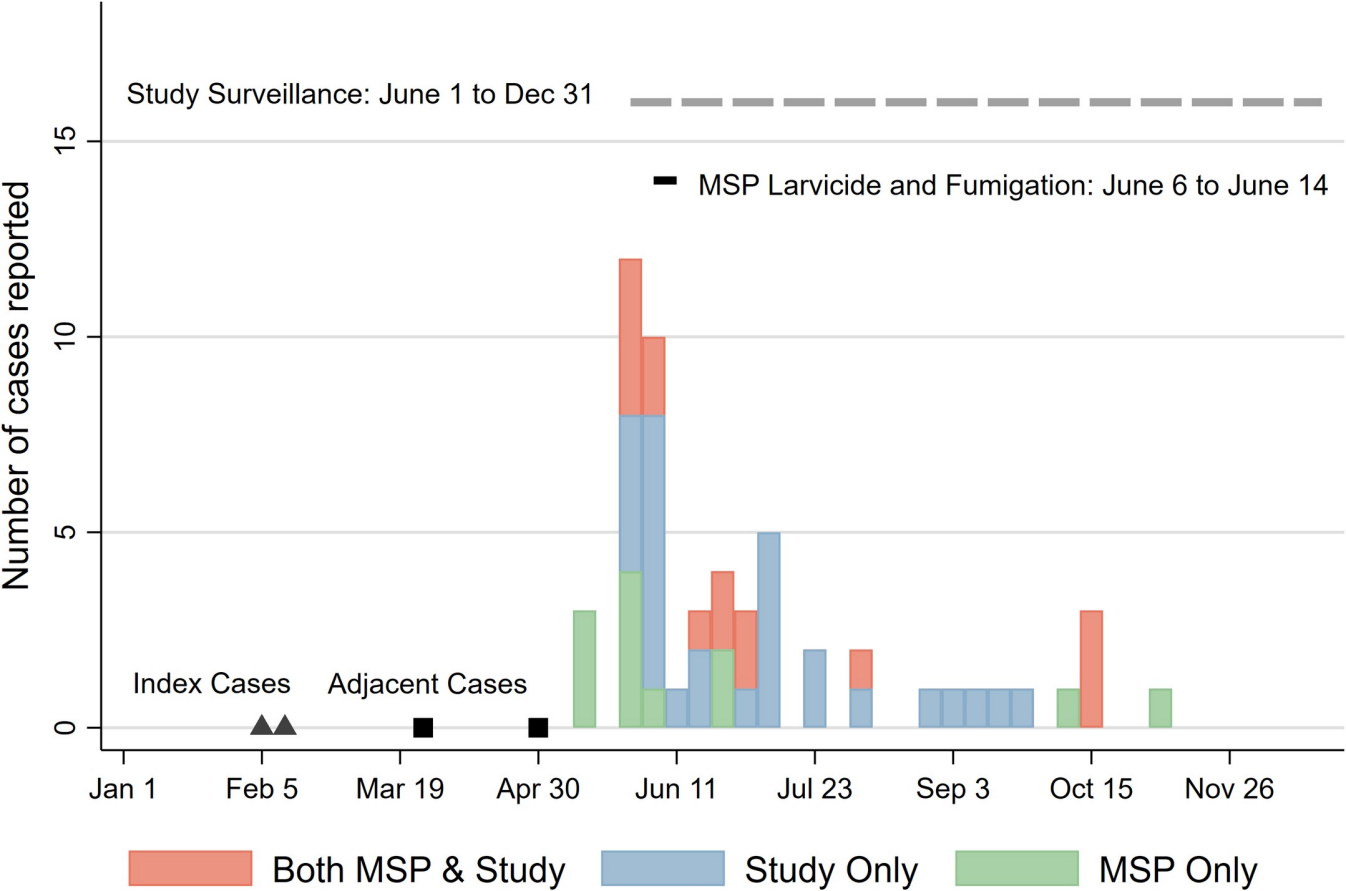
Cases identified by reported date of fever onset: Cases identified during the 2019 calendar year. Suspected index cases are represented by triangles, while adjacent cases (one from a person living in a nearby community, and one from a person diagnosed after returning from a trip to the village) are represented by square. From Jan 1^st^ to May 31^st^, case detection was based on Ministry of Health (MOH) clinical records, from Jun 1^st^ to December 1^st^, case detection was based of combined MOH and community-based active surveillance.

The suspected index cases were two young people from the community, who had been studying in the city of Esmeraldas and visited the village during the school holidays in February. The students were diagnosed on February 14^th^ and February 20^th^, respectively, with symptoms beginning on February 9^th^ and 13^th^. In both instances, the infection was thought to have occurred in the city of Esmeraldas. On March 28^th^, the health post identified a clinically suspected case in a person who lived in a community nearby and on May 2^nd^, a secondary school student visited the village and was diagnosed with clinically suspected dengue after returning to their home in Quito. All cases reported by community residents occurred from mid-May 2019 onward (**Fig 3**). From May 15th to November 8th, 55 symptomatic cases were identified in 37 unique households, representing 9.5% of the total population (576 individuals in 128 households). Twenty-seven (27) households had one case, five households had two cases, three households had three cases, one household had four case, and one household had 5 cases, for a secondary attack rate of 0.133. Twelve cases (12) were identified by the local clinic only (mostly before our active surveillance began in June 2019), 28 were identified by our active surveillance only, and 15 were identified by both our active surveillance and the local clinic (**Fig 3**). All laboratory confirmed cases were dengue virus serotype 1 (DENV-1). Of the 27 cases identified by the local clinic, 22 were without warning signs, and 5 with warning signs. Of the 43 cases identified by study active surveillance, 22 reported at least one severe symptom (abdominal pain, vomiting, difficulty breathing, mucosal bleeding, lethargy, or restlessness), although this is not equivalent to a clinical case definition of dengue with warning signs. In response to these cases, the MOH fumigated and distributed larvicide to all households in early June (**S1 Table**).

### Entomology survey

Based on the entomological survey that took place on May 18^th^, the most common type of larvae or pupae containing container were larger 55-gallon tanks or rain barrels (58.7%). These containers are also the most readily identifiable by UAV. However, less than half of all positive containers (38.8%), and larger positive containers (47.1%) were located outside of the household. The overall community Breteau index was 18.2 (10 positive containers per 55 households inspected). When calculated separately for Indigenous and Afro-Ecuadorian households, the Breteau index was 4.5 for Indigenous households, and 20.0 for Afro-Ecuadorian households (**Table 1**). A total of 11 adult *Ae*. *aegypti* were captured in four of 55 households (**Fig 2**). Two of these were Afro-Ecuadorian households, one was a Chachi household, and one household could not be ascertained because it had been uninhabited at the time the community was censused, when ethnicity was documented. One pooled sample of adult female mosquitos was found to be positive for DENV-1.

**Table 1:** Comparison of Afro Ecuadorian and Chachi Households. Four households that described themselves as mestizo were excluded.

	Afro Ecuadorian Households	Chachi Households	
	N = 69	N = 55	p-value
**Survey variables:**			
**Household size (mean (SD))**	3.3 (1.9)	5.7 (2.7)	<0.001
**Household education**	12.4 (3.2)	10.9 (4.3)	0.0435
**Roof material:**			
**zinc** *	98.4%	96.0%	0.437
**other material**	1.6%	4.0%	
**Floor material:**			
**cement**	53.2%	18.0%	<0.001
**wood**	19.4%	80.0%	
**other**	27.4%	2.0%	
**Wall material:**			
**cement**	79.0%	71.4%	<0.001
**wood**	12.9%	28.6%	
**other**	8.1%	0.0%	
**Primary water for domestic use:**			
**rain**	87.1%	76.0%	0.2230
**river**	12.9%	22.0%	
**other**	0.0%	2.0%	
**Spatial variables:**			
**Local population density (# of people living in 40m buffer)**	38.4 (18.0)	59.0 (24.1)	<0.001
**# of containers per household identified by UAV**	1.7 (0.3)	1.2 (2.0)	0.1668
**# of containers identified by drone within 40m buffer of household**	16.8 (10.3)	12.6 (7.4)	0.0135
**Local GRVI**	0.07 (0.03)	0.05 (0.05)	0.0052
**Entomological variables:**			
**Households participating in entomological survey** **	30	22	n/a
**# of containers per household identified by entomological survey** **	4.2 (2.7)	5.0 (3.0)	0.3311
**Breteau Index** **	20.0 (61.0)	4.5 (21.3)	0.3755

*62 Afro HHs and 50 Chachi HHs assessed in the HH survey

**calculated based on 55 households: 30 Afro-Ecuadorian, 22 Chachi, 2 Mestizo/Manabi, 1 household in which ethnicity could not be ascertained.

### Spatial risk factors for symptomatic dengue

**Fig 4:**
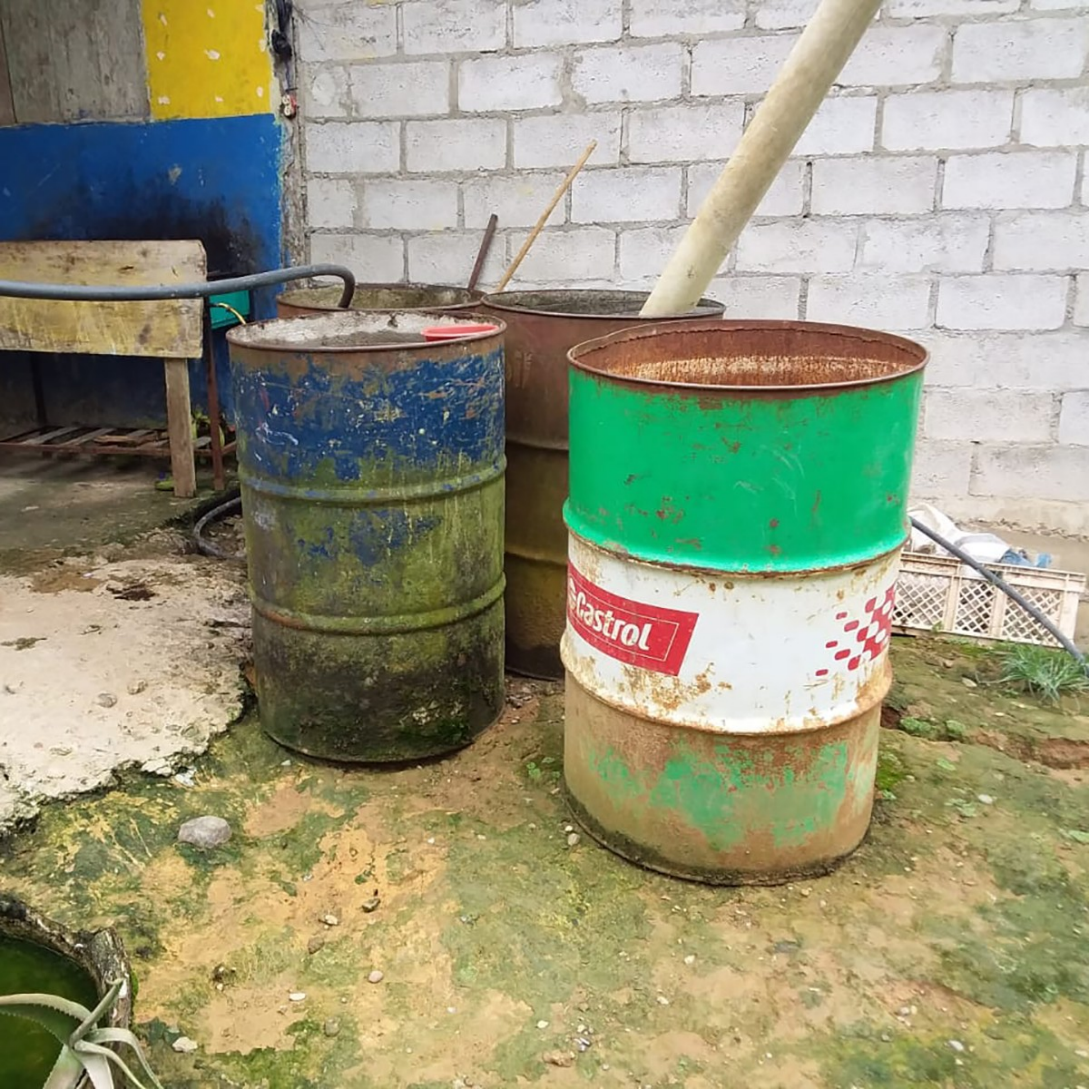
Outdoor rain barrels: A photo of an outdoor rain barrel.

**Fig 5:**
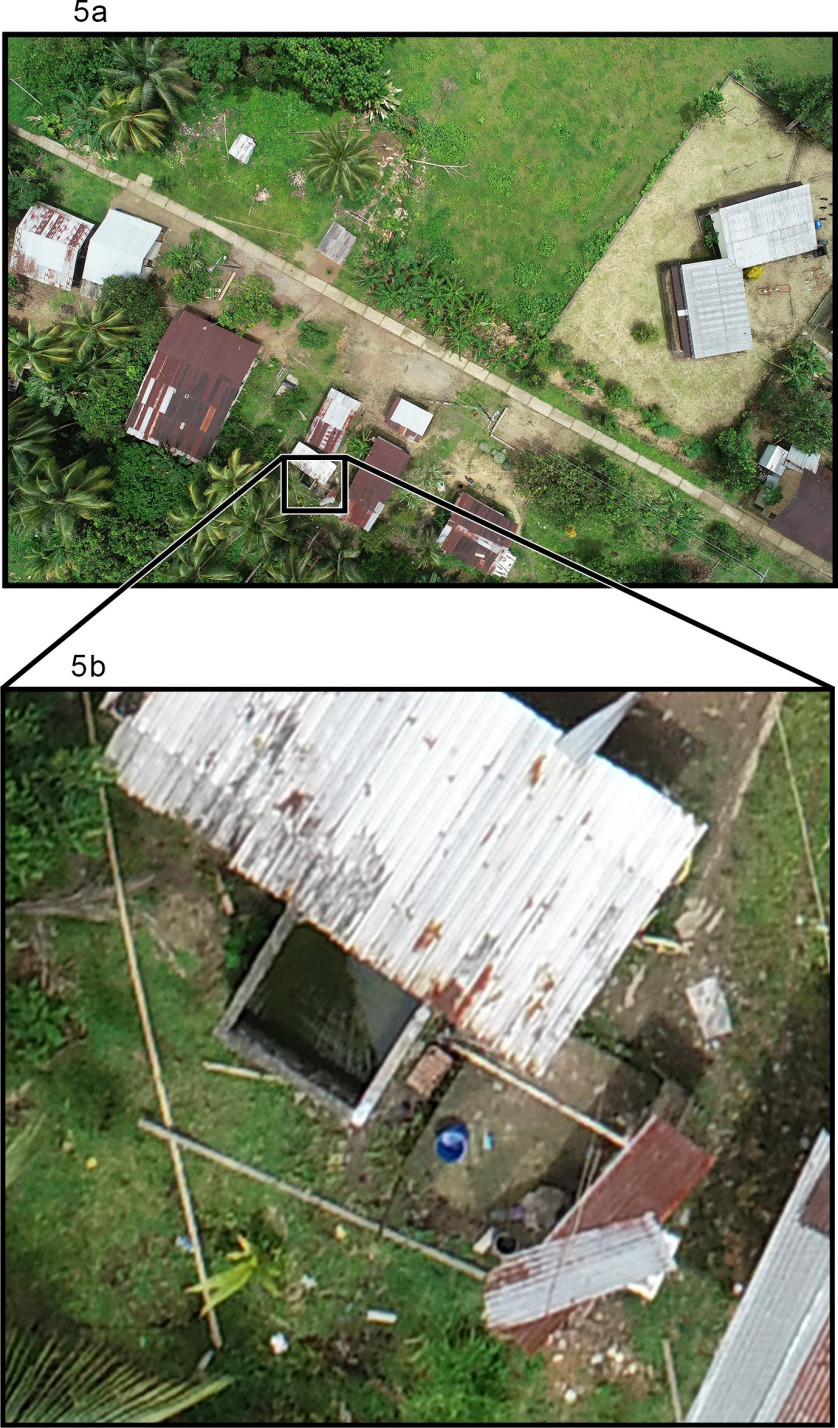
**UAV images**: Larger, outdoor potential larval habitats such as cisterns and rain barrels can be identified from UAV images (Fig 5A and 5B).

The overall green-red vegetation index (GRVI) was 0.064 (95%CI: 0.059, 0.071). Potential values of the GRVI range from -1 to 1, with higher values representing greater vegetation. A GRVI of zero represents a threshold that discriminates between canopy cover and other conditions (i.e., bare soil or water) [28]. We identified 262 unique containers by UAV, which are potential *Ae*. *aegypti* larval habitats. Some (22) of these containers were not associated with any household but rather with public buildings, and a further 29 were associated with temporarily uninhabited or abandoned homes. Examples of containers are shown in **Figs 4** and **5**. Based on visual inspection of images, an average of 1.58 (95%CI: 1.18, 1.99) containers per household were identified while an average of 14.78 (95%CI: 13.18, 16.78) containers were identified within 40m of each household. Among the subset of 55 households that participated in the entomological survey, the average number of containers identified per household, based on the visual confirmation of the surveyor, was 4.60 (95%CI: 3.79, 5.40). There was no correlation between the number of UAV-identified containers and the number of entomologist-confirmed potential larval habitats associated with any household (Spearman’s rho = -0.05, p = 0.7097), and only around a third (35.00%) containers surveyed by entomologists were likely identifiable by UAV based on having a size of 5 liters or greater and being located outdoors.

Simple logistic regression analyses suggested that cases were more likely to be of Afro-Ecuadorian ethnicity and were geographically clustered on the eastern side of the community. Chachi households were larger than Afro-Ecuadorian households, and there was a higher population density in the Chachi (riverfront) versus the Afro-Ecuadorian side of the community (59.0 versus 38.4 individuals living within 40m of a household, p<0.001) (**Table 1**). Chachi households also had relatively lower vegetation indices (mean GRVI of 0.05 versus 0.07, p = 0.0052) compared to Afro-Ecuadorian ones. Additionally, Chachi households had, on average, fewer containers within a 40-meter buffer than Afro-Ecuadorian households (12.6 versus 16.8, two-sided t-test p = 0.0135). Overall, 24.0% of Chachi households reported that their primary source of water for domestic use was the river, versus 12.9% of Afro-Ecuadorian (p = 0.1297). For both groups, the main water source was collected rainwater (**Table 1**).

In multivariate mixed effects logistic regression models, age, ethnicity, living within 40m of the town football field, and having a higher local GRVI, were statistically significantly associated with symptomatic dengue (**Tables 2 and 3**). Compared to the crude unadjusted estimates, the relationship between ethnicity and symptomatic dengue was attenuated in the adjusted model (OR = 2.99 in unadjusted model versus 1.00 in adjusted model). Adolescents and adults age 15–30 and adults age 30–60 were at greater risk compared to the reference group of children 0–15 (OR = 2.56, 95%CI: 1.05, 6.28, p = 0.039 and OR = 2.48, 95%CI: 1.03, 5.91, p = 0.041, respectively). Sex, reported livelihood, and the number of UAV-identified containers within 40m was not associated with symptomatic dengue (**Table 3**). Statistical analyses were completed in qGIS version 3.2.1 [30] and Stata version 15.0 [31].

**Table 2:** Characteristics of Cases.

	Cases	Non-Cases	p-value
**Ethnicity:**			
Afro-Ecuadorian*	61.8% (N = 34)	37.6% (N = 196)	0.003
**Chachi**	30.9% (N = 17)	55.5% (N = 289)	
**Mestizo**	7.3% (N = 4)	6.9% (N = 36)	
**Age (mean (SD))**	30.3 (2.6)	28.7%	0.5685
**Male sex**	49.1%	50.3%	0.8662
**Self-reported prior dengue** *	1.9%	6.3%	0.1862
**Livelihood:**			0.737
**Child < = 13 years old**	23.6% (N = 13)	31.9% (N = 166)	
**Housewife**	40.0% (N = 22)	31.7% (N = 165)	
**Agriculture or physical labor**	21.8% (N = 12)	20.7% (N = 108)	
**Government employee, including teacher & healthcare worker**	9.1% (N = 5)	7.7% (N = 40)	
**Small business owner**	1.8% (N = 1)	2.3% (N = 12)	
**Other**	3.6% (N = 2)	5.8% (N = 30)	

*N = 545, 31 = “Don’t know”

**Table 3:** Mixed Effect Logistic Regression: Factors associated with case status. Risk factors for symptomatic dengue based on bivariable and multivariable mixed-effect logistic regression models.

	Unadjusted Models		Adjusted Models	
	Odds Ratio (95% CI)	p-value	Odds Ratio (95% CI)	p-value
**Sex (Ref: Female):**				
**Male**	1.09 (0.58, 2.06)	0.793	-	
**Ethnicity (Ref: Chachi):**				
**Afro-Ecuadorian**	2.99 (1.29, 6.92)	0.011	1.00 (0.42, 2.36)	0.999
**Other (mestizo/manabi)**	1.87 (0.44, 7.88)	0.394	1.16 (0.31, 4.43)	0.823
**Age (Ref: Age < = 15 years):**				
**15–30 years**	2.40 (0.99, 5.86)	0.054	2.56 (1.05, 6.28)	0.039
**30–60 years**	2.11 (0.92, 4.84)	0.077	2.48 (1.03, 5.91)	0.041
**>60 years**	1.63 (0.41, 6.46)	0.049	1.80 (0.48, 6.79)	0.383
**Livelihood (Ref: Child< = 13 years)**				
**Housewife**	2.22 (0.95, 5.19)	0.066	-	-
**Agriculture or physical labor**	1.77 (0.68, 4.65)	0.243	-	-
**Government employee**	2.08 (0.56, 7.67)	0.272	-	-
**Small business owner**	1.86 (0.16, 21.1)	0.617	-	-
**Other**	1.07 (0.18, 6.22)	0.941	-	-
**Household size**	0.96 (0.81, 1.14)	0.674	-	-
**Household education**	0.92 (0.81, 1.04)	0.179	-	-
**Roof material (ref: zinc)**				
**other**	0.34 (0.02, 6.15)	0.465	-	
**Flood material (ref: cement)**				
**wood**	0.30 (0.11, 0.78)	0.014	-	
**other**	0.35 (0.09,1.40)	0.138	-	
**Wall material (ref: cement)**				
**wood**	0.48 (0.18, 1.24)	0.130	-	
**= other**	2.36 (0.23, 24.60)	0.472	-	
**Water source (ref: rainwater)**				
**river**	0.63 (0.18, 2.23)	0.473	-	
**Local population density**	0.98 (0.96, 0.99)	0.020	-	
**Local container density**	1.01 (0.96, 1.06)	0.674	-	
**Local GRVI (per 0.10 increase)**	6.14 (1.85, 20.36)	0.003	11.33 (0.38, 38.0)	<0.001
**Lives within 40m of football field**	11.11 (3.7, 33.29)	<0.001	13.94 4.02, 48.36)	<0.001

*Highest number of years of education completed by any member of the household

## Discussion

In 2019, almost ten percent of the study village experienced a symptomatic DENV infection. Although the study village is connected to the rest of Ecuador through human movement, there are no direct roads and almost all access occurs through a single entry point, the town river port. Given that most of the population was stationary, the clustering of symptomatic dengue cases within households and neighborhoods suggests that, following the two index cases, disease was acquired within the community, rather than through multiple importation events. The overall community Breateau index was also well above the estimated threshold to sustain transmission [32]. Despite an early chemical-based vector control response by the MOH, transmission likely continued for several months.

Since the suspected index cases for this outbreak occurred in February, and two ‘adjacent’ cases (one visitor and one resident of a nearby community) occurred in March and early May, it is likely that the first cases of dengue identified among residents in mid-to-late May were third or fourth ‘generation’ infections. Although this study benefits from a strong epidemiological surveillance system operated by the MOH, which was active throughout 2019, we are also limited by a lack of community-based active case detection in the first half of the year and the lack of information on asymptomatic infections.

Given the shifting and intensifying epidemiology of dengue in rural Latin America, there is a need to understand risk factors within rural communities. We utilized multiple data streams, including UAV maps, entomological data, active fever surveillance and MOH case capture to identify risk factors for symptomatic infection. Several differences in the physical environment and in behavior between the two sides of the community were identified that may have driven these differences in risk. On the one hand, Afro-Ecuadorian households had some features that might suggest they were at lower risk compared to the Indigenous Chachis. For example, they were more likely to have homes constructed of cement and had lower local population density. On the other hand, Chachi households had fewer nearby water containers identifiable by UAV, a difference likely driven by a slightly greater preference for river water over rainwater and reduced distance from the river, resulting in less water storage. Proximity to a river has also been associated with decreased dengue risk in other contexts [33]. However, in multivariate logistic regression models, denser vegetation and proximity to the town football field were more robust predictors of symptomatic dengue than proximity or distance to the river. Anecdotally, community members working with the study identified a temporarily uninhabited house at the corner of the football field that may have been an important larval habitat. Proximity to the football field, a site of congregation, may also help to explain why adolescents and younger adults were at greatest risk of disease. The inclusion of vegetation in the model caused the relationship between ethnicity and dengue to disappear, suggesting that this aspect of the physical landscape best explained the differences between the two groups. Overall, our results suggest that some transmission occurred within the perimeter of a case’s home, and some transmission may have occurred during group events, such as football matches, but the lack of data on movement within the community limits our ability to fully disaggregate these two mechanisms.

We used UAVs to characterize fine-scale heterogeneity within the community landscape, an approach useful in identifying the individual and joint risk factors that cumulatively produced an environment ecologically suited to promote *Ae*. *aegypti* infestation and dengue transmission. In conducting our analysis, we identified features of UAV data that should be taken into consideration when performing future analyses using this type of data. First, UAV-identified containers were not a good proxy for the presence of container larval habitats at the household level. This is not surprising, as it was generally not possible to differentiate properly covered containers, versus containers with no screen or cover, from the UAV image. In addition, many containers are small and/or kept indoors, or under the eaves of the household, where they are not identifiable from an aerial view. On the other hand, the UAV approach was useful in characterizing neighborhood-level differences in the number of containers present, and in identifying containers that were associated with public buildings rather than households and would therefore have been overlooked by household surveys only. As others have noted [13], UAV images were also useful to qualitatively understand the community landscape and may have utility as a method to identify features that may not be recognized, *a priori*, as points of interest. In this instance, the football field represents such a feature. Finally, we found that the green-red vegetation index was associated with disease risk. While vegetation is an informative indicator to extract from an UAV image, future work should aim to develop machine-learning classification approaches to better characterize other key environmental features. To shift UAV mapping from a useful research tool into a tool for programmatic outbreak management, future work should aim to establish the extent to which UAV-identifiable features may be sensitive and specific indicators of vector density.

Our study has several limitations, including the lack of community-based active febrile surveillance in the first half of 2019, and lack of within-community human movement data, as described above. Our entomological surveillance characterized only larval habitat containers, or one randomly selected negative container per household, limiting our ability to test whether UAV mapping is a good proxy for large, outdoor containers, as opposed to containers overall. We are also limited by a lack of data on asymptomatic infections, which may represent 52–88% of cases [34–37]. While we do not have access to dengue seroprevalence data prior to the epidemic, in late 2019, at the end of the epidemic, we conducted a serosurvey during which we estimated 79% of the community was positive for dengue IgG antibodies. Previously, we had also estimated the prevalence of prior Zika infection by Blockade of Binding ELISA [38,39] in the community to be 18%. Given cross-reactivity between DENV and Zika IgG ELISA assays, these results suggest that between 57–75% of the population had been exposed to dengue at some time.

We are further also limited by the fact that this outbreak took place in a relatively small community. Furthermore, UAV mapping was conducted only once, at the beginning of the study. Although we observed associations between some UAV-identified features and symptomatic dengue, we were likely underpowered to fully characterize all potentially relevant risk factors. Future work could aim to use repeated UAV flights to map changing landscapes prospectively, during epidemics: a low-cost method of generating high-resolution temporal and spatial data.

On the other hand, a strength of our approach is the combination of UAV mapping with other innovative data streams available through this project, resulting in rich and high-quality data even in a rural and still relatively isolated community. Future work may aim to implement UAV mapping through participatory community partnerships. Proof-of-concept for this approach has already been demonstrated by an ongoing project operating in Ecuadorian Amazonian communities, in which community members were trained to use UAVs, as well as other technologies, to monitor difficult-to-access community-controlled territory [40]. Community-based monitoring systems that bring technology to communities for citizen science are being experimented within the fields of geography and environmental science. For public health practitioners and epidemiologists, partnering with communities to collect surveillance data, as represented by our ‘brigadista’ model, is also a powerful model for data collection, as are ‘task shifting approaches’ that put data collection usually relegated to specialists into the hands of community members. To support empowerment and capacity building, these partnerships should not be limited only to data collection but should seek adopt community-based participatory research approaches by engaging community partners in dissemination and interpretation the results of these efforts.

## Conclusion

Heterogeneity in the physical environment and water storage practices, and thus dengue risk, exists even within a small rural community. Combining UAV mapping with other data enhanced our ability to identify, with high resolution, variations in the community environment related to disease risk. This complementary approach informs our understanding of DENV transmission dynamics in rural areas.

## Supporting information

S1 Table Vector Control Activities. Vector control activities undertaken by the MOH in response to the outbreak.
(DOCX)

S2 Table Associations between dengue status and key features in community. Results of logistic mixed regression models with a random incept to account for Household-level clustering.
(DOCX)
