# Supplementary material for: A dengue outbreak in a rural community in Northern Coastal Ecuador: An analysis using unmanned aerial vehicle mapping
Source: PLoS Negl Trop Dis. 2021 Sep 27;15(9):e0009679. doi: 10.1371/journal.pntd.0009679 (PMC8475985; doi:10.1371/journal.pntd.0009679)
Supplement: S1 Table — Vector control activities undertaken by the MOH in response to the outbreak. (DOCX) [file pntd.0009679.s001.docx]

**Supplemental Table 1: Vector Control Activities**

| **Date** | **Activity** | **Reason for activity** | **Part of community** | **Coverage** |
| --- | --- | --- | --- | --- |
| June 6^th^ | Fumigation | Reported case | Afro-Ecuadorian side | - - 54HHs fumigated with Deltamethrin   - 24 vacant lots fumigated with Malathion |
| June 7^th^ | Fumigation | Reported case | Chachi side | - - 92HHs fumigated with Deltamethrin |
| June 11^th^ | Larvae control | Reported case | Afro-Ecuadorian side | - - 60 HHs screened   - 4 vacant lots screened   - 289 reservoirs screened   - 289 reservoirs treated |
| June 12^th^ | Fumigation | Reported Case | Afro-Ecuadorian side | - - 61HHs fumigated with Deltamethrin |
| June 13^th^ | Larvae control | Routine | Chachi side | - - 84 HHs screened   - 5 vacant lots screened   - 331 reservoirs screened   - 323 reservoirs treated |
| June 14th | Fumigation | Reported Case | Chachi side | - 62HHs fumigated with Deltamethrin - 24 vacant lots in Camarones fumigated with Malathion |
