# Supplementary material for: A dengue outbreak in a rural community in Northern Coastal Ecuador: An analysis using unmanned aerial vehicle mapping
Source: PLoS Negl Trop Dis. 2021 Sep 27;15(9):e0009679. doi: 10.1371/journal.pntd.0009679 (PMC8475985; doi:10.1371/journal.pntd.0009679)
Supplement: S2 Table — Results of logistic mixed regression models with a random incept to account for Household-level clustering. (DOCX) [file pntd.0009679.s002.docx]

**Supplemental Table 2:** associations between dengue status and key features in community

**Caption:** Result of logistic mixed regression models with a random incept to account for Household-level clustering.

|  | **Odds Ratio (95% Confidence Interval)** | **p-value** |
| --- | --- | --- |
| **Football field** | 11.11 (3.7, 33.29) | <0.001 |
| **High school*** | - | - |
| **Elementary school** | 0.42 (0.11, 1.64) | 0.215 |
| **Nursery school** | 0.12 (0.01, 1.45) | 0.096 |
| **Church*** | - | - |
| **Info center** | 1.74 (0.40, 7.41) | 0.460 |
| **Piragua 1** | 0.09 (0.01, 0.99) | 0.049 |
| **Piragua 2*** | - | - |
| **Piragua 3** | 0.34 (0.05, 2.45) | 0.284 |
| **Piragua 4** | 1.63 (0.24, 11.10) | 0.615 |
| **Police Station** | 6.48 (1.71, 24.50) | 0.006 |
| **Health Center** | 2.27 (0.49, 10.45) | 0.292 |
| **River** | 0.25 (0.10, 0.63) | 0.003 |

*No cases occurred near these sites, odds ratios could not be estimated
